# Efficacy and Safety of Valproic Acid Transition Regimens from Intravenous to Oral Administration in Epileptic Patients: A Single-Center Cross-Sectional Study

**DOI:** 10.3390/jcm14207442

**Published:** 2025-10-21

**Authors:** Liying Chen, Yiting Zhou, Jing Zhang, Lisan Zhang, Guodong Lou

**Affiliations:** 1 Department of Pharmacy, Sir Run Run Shaw Hospital, School of Medicine, Zhejiang University, Hangzhou 310016, China; 3320057@zju.edu.cn (L.C.); zyt1567@zju.edu.cn (Y.Z.); 3415340@zju.edu.cn (J.Z.); 2 Department of Neurology, Sir Run Run Shaw Hospital, School of Medicine, Zhejiang University, Hangzhou 310016, China; zls09@zju.edu.cn

**Keywords:** epilepsy, valproic acid, dosage form transition, efficacy and safety, adverse drug reactions, blood drug concentration

## Abstract

**Objectives**: This study aims to evaluate the efficacy and safety of valproic acid (VPA) transition regimens (from intravenous to oral tablets) for anti-seizure treatment. **Methods**: A retrospective analysis was conducted on inpatients treated with intravenous VPA and oral tablets for epilepsy at the Sir Run Run Shaw Hospital, affiliated with Zhejiang University, between January 2022 and December 2023. Various transition strategies from VPA injections to tablets were examined, and the efficacy and safety of different transition strategies were analyzed. **Results**: A total of 164 inpatients receiving VPA transition therapy were included in this study, which was divided into three groups based on the transition timing: the 0 h group, the 0–48 h group, and the >48 h group. Regarding VPA dosage, the median daily dose of intravenous VPA was separately 1076.50 mg/day, 1200 mg/day and 1438 mg/day in the 0 h group, 0–48 h group, and the >48 h group. During transition, the daily doses of VPA were significantly higher than that before and after the transition. After completely switching to oral administration, they were all decreased to 1000 mg/day. Moreover, a significant difference regarding the clinical efficacy was observed among the three groups. The >48 h group showed the highest rate of clinical efficacy, which was significantly greater than that of the 0 h group and 0–48 h group. Although there was no statistical significance detected regarding the average blood serum concentrations among the three groups; notably, a higher proportion of patients in the >48 h group (19.35%) had blood concentrations exceeding the desired therapeutic window compared with the 0–48 h group (8.06%) and 0 h group (0%). Adverse events included 30 cases in the 0 h group, 42 in the 0–48 h group, and 67 in the >48 h group, with statistically significant differences in hemoglobin reduction, headache/dizziness, and liver injury. No significant differences were found in digestive and skin-related reactions. **Conclusions**: The results suggest that the >48 h transition regimen may show some advantages in efficacy but also increases the risk of adverse reactions significantly. Therefore, it is recommended to complete the intravenous-to-oral switch carefully with blood drug concentrations strictly monitored.

## 1. Introduction

Epilepsy is a chronic neurological disorder characterized by recurrent, transient dysfunction of the central nervous system (CNS) caused by abnormal, excessive, and synchronous neuronal discharges in the brain [1,2]. According to estimates from the World Health Organization, around 50 million people worldwide are affected by epilepsy, with more than 4 million new cases reported annually [3]. Anti-seizure medications (ASMs) remain the most fundamental and crucial method for epilepsy management, effectively suppressing seizures in up to 2/3 of epilepsy patients, although they do not prevent or reverse epileptogenesis [4,5]. Among ASMs, valproic acid (VPA) is widely recommended by clinical guidelines as a first-line treatment for multiple types of seizures, including generalized tonic–clonic, myoclonic, and absence seizures. In addition to being a therapeutic intervention in clinical practices, it is also commonly used for seizure prevention. However, the use of VPA requires careful monitoring due to its narrow therapeutic window and the potential dose-dependent adverse effects [6,7]. While most adverse drug reactions (ADRs) are mild and reversible upon discontinuation of the medication, such as dizziness and sleepiness, more severe reactions can occur, making it crucial to thoroughly assess its biosafety in clinical applications.

VPA has been available as an injectable formulation since 1993. In cases such as status epilepticus (SE) and refractory generalized seizures, intravenous (IV) administration of VPA is commonly applied due to its rapid onset, allowing for the quick attainment of therapeutic levels and effective seizure control [8,9]. However, for long-term management, a timely transition from intravenous to oral VPA (IV-to-oral transition) is recommended when clinically appropriate. This switch helps to reduce the risks associated with prolonged IV use, such as higher costs and extended hospital stays. In China, it is generally recommended that the transition from IV to oral should be conducted gradually, with brief overlapping (no more than 48 h) to avoid sudden drops in blood drug concentrations. However, currently, there are no standardized guidelines for the transition, which can make clinical decision-making more challenging. In practice, the decision is often made according to the attending physician’s personal clinical experience. Thus, this switch is often achieved with various strategies, including an immediate transition from IV to oral VPA after discontinuation of the IV form, or a combined IV-oral regimen, where the IV dose is gradually reduced over several hours to days before completely transitioning to oral therapy.

On the one hand, an immediate transition may cause a robust reduction in steady-state VPA plasma concentrations, potentially resulting in inadequate seizure control or even recurrence. On the other hand, prolonged combination therapy may lead to excessively high daily doses of VPA, pushing blood levels beyond safe thresholds and thereby increasing the risk of adverse events. Both of these scenarios pose serious health risks and can result in irreversible harm to patients. Therefore, further studies to optimize strategies for the IV-to-oral switch of VPA is necessary to ensure effective epilepsy control and enhance medication safety.

This study retrospectively collected and analyzed data from epilepsy inpatients treated with VPA injections and tablets, focusing on the strategies employed for transitioning from IV to oral VPA, evaluating the efficacy and safety of different switching strategies. By providing insights into various transition methods, this cross-sectional study is intended to offer practical guidance for safe and effective clinical management of the IV-to-oral VPA transition.

## 2. Materials and Methods

### 2.1. Subjects

**Figure 1:**
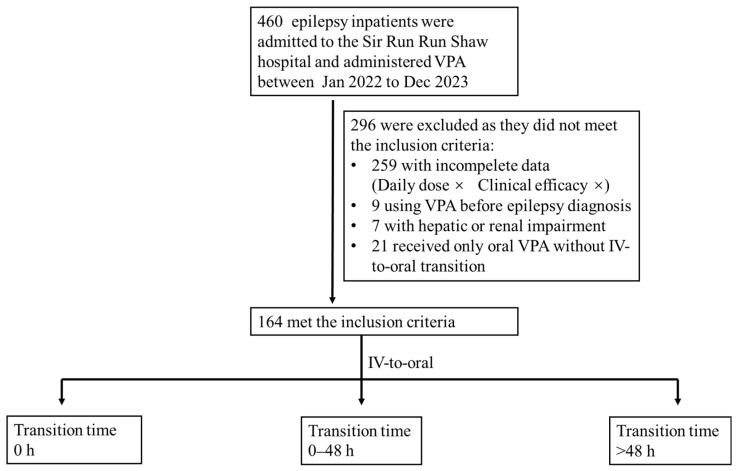
Flowchart of patient selection, exclusion, and grouping.

A single-center retrospective analysis was conducted on adult epilepsy inpatients at Sir Run Run Shaw Hospital, Zhejiang University. Epilepsy patients who received intravenous VPA treatment and underwent an IV-to-oral switch during hospitalization, between January 2022 and December 2023, were included. Patients were excluded if they met any of the following criteria: (1) prior use of VPA for non-epileptic conditions or before their epilepsy diagnosis; (2) hepatic or renal impairment; (3) or incomplete clinical data. Epileptic seizures were identified by specialized clinicians (neurologists/epileptologists) and documented in the patients’ medical records. They identified and defined an epileptic seizure based on: (1) ambulatory EEG monitoring (AEEG) recordings; (2) the clinical symptoms described and reported by patients, their family members, and nursing staff (Figure 1).

### 2.2. Study Design

The study received approval from the Ethics Committee of the Sir Run Run Shaw Hospital, School of Medicine, Zhejiang University (2025-2314-01, 24 April 2025) and was conducted according to the ethical guidelines of the Declaration of Helsinki. The requirement for written informed consent for this retrospective study was waived by the internal review board of the Sir Run Run Shaw Hospital, School of Medicine, Zhejiang University.

Data on epilepsy patients prescribed VPA during hospitalization were extracted from the database of Sir Run Run Shaw Hospital, Zhejiang University, spanning January 2022 to December 2023. Prior to extraction, all researchers jointly reviewed and reached a consensus on the predefined inclusion/exclusion criteria, finalizing a standardized extraction protocol to ensure consistency. Two trained researchers then independently extracted data using a structured Excel form (with pre-defined fields for key variables, e.g., seizure frequency, VPA dose). After initial extraction, they cross-validated their records—any discrepancies (e.g., conflicting seizure count documentation) were resolved by consulting original medical records and a senior neurologist (L.S.Z.) not involved in extraction process.

Based on the transition time from IV-to-oral VPA, the patients were finally categorized into 3 groups: the 0 h group, the 0–48 h group, and the >48 h group. The efficacy and safety of different IV-to-oral switching strategies were assessed based on the following observational indicators.

#### 2.2.1. Observational Indicators

Clinical efficacy: the frequency of seizures before, during, and after the IV-to-oral transition.

Laboratory test indicators: liver function parameters (e.g., ALT, AST, ALP), hematological parameters (e.g., hemoglobin, white blood cell count, platelet count), and blood plasma concentrations of VPA.

As a retrospective study, the criteria for determining the timing of blood sampling for plasma drug concentration were relatively lenient. Therapeutic drug monitoring (TDM) was not performed according to predefined pharmacokinetic principles. Instead, TDM was often conducted on an “as-needed” basis rather than at consistent time points relative to efficacy/safety assessments.

Generally, the blood sampling timepoints for three phases of VPA transition were separately defined, as follows:

(1) Pre-transition phase: Blood samples were collected 48–72 h after the initiation of intravenous VPA (after intravenous VPA reached steady state).
(2) During transition: Blood samples were typically collected 24–48 h after the initiation of the transition. Additional sampling was performed after 72 h if the transition had not been completed by 48 h (for the >48 h group). However, since clinicians often adjust the doses of intravenous and oral VPA based on their clinical experience (with the dosage of intravenous VPA decreasing and the dosage of oral VPA increasing), with variations in the timing of these dose adjustments, it is challenging to define and monitor the steady-state plasma concentration of VPA at this phase in patients. Thus, the measured plasma concentrations can only roughly reflect the plasma drug level during the transition phase.
(3) Post-transition phase: Blood samples were collected after 72 h (after oral VPA reached steady state).

Adverse reactions: observed treatment-related reactions, including gastrointestinal reactions (e.g., nausea, vomiting, abdominal pain, diarrhea), neurological reactions (e.g., drowsiness, headache, dizziness, mood changes), and allergic reactions (e.g., rash, pruritus).

#### 2.2.2. Efficacy Evaluation

The efficacy of treatment was assessed and then categorized into 3 levels: “Marked efficacy”, “Efficacy”, “Inefficacy”. (1) Marked efficacy: This represents the highest level of therapeutic success, where the patient’s seizure frequency is reduced by more than 75%. (2) Efficacy: This level indicates a moderate therapeutic effect, where the seizure frequency is reduced by 50–75%. (3) Inefficacy: This category denotes a lack of significant therapeutic effect, with a reduction in seizure frequency of less than 50%; in some cases, symptoms might even worsen.

The total efficacy rate was calculated as follows:

$$\mathrm{Total}\;\mathrm{efficacy}\;\mathrm{rate}\;(\%)\;=\;\frac{Number\;of\;cases\;with\;marked\;efficacy+Number\;of\;cases\;with\;efficacy}{Total\;number\;of\;cases}\;\times \;100\%$$

#### 2.2.3. Adverse Reaction Evaluation

The Naranjo Scale was utilized to assess the probability of ADRs [10,11]. This scale consists of 10 objective questions, which help evaluate the likelihood of a drug causing an adverse reaction. Based on the answers to these questions, a probability score is calculated, and the causality of the ADR is then determined according to the resulting score. The higher the score, the stronger the evidence suggesting that the drug is responsible for the ADR. Based on the total score, ADR causality was classified into 4 categories: Definite (≥9), Probable (5–8), Possible (1–4), Doubtful (≤0) (Table 1).

### 2.3. Statistics

Statistical analyses were conducted with SPSS 26.0 software. Continuous variables were assessed for normality of distribution with Shapiro–Wilk test. Quantitative data that followed a normal distribution were presented as mean ± SEM and analyzed using One-Way ANOVA. Non-normally distributed quantitative data were expressed as median (interquartile range) and analyzed using Kruskal–Wallis tests or Mann–Whitney tests. Categorical data were expressed as counts (%) and analyzed using χ^2^ tests or Fisher’s exact tests. A *p*-value < 0.05 was considered statistically significant. For multiple comparisons, *p* values Bonferroni corrected were applied.

## 3. Results

### 3.1. Study Population Characteristics

The study included data from 164 epilepsy patients, between January 2022 and December 2023. Based on the transition time from IV-to-oral VPA, the patients were categorized into three groups: the 0 h group (40 cases), the 0–48 h group (62 cases), and the >48 h group (62 cases). There were no significant differences in gender distribution across the three groups: 0 h group (24 males, 60%), 0–48 h group (43 males, 69.4%) and >48 h group (45 males, 72.6%). Similarly, no significant differences were found in age distribution: 0 h group (25 patients < age 65, 62.5%), 0–48 h group (33 patients < age 65, 53.2%) and >48 h group (37 patients < age 65, 59.7%). In addition, there was no significant difference in the history of epilepsy regarding seizure type (generalized seizure, GS vs focal seizure, FS) among the groups: 0 h group (GS, 50%), 0–48 h group (GS, 53.2%) and >48 h group (GS, 54.8%) (Table 2). No significant difference in seizure type was observed among the groups (*p* = 0.891). The majority of patients were admitted from the Neurology (60 cases, 36.6%) and Neurosurgery (57 cases, 34.8%) departments.

### 3.2. Length of IV VPA Before Switching

We extracted the duration of IV VPA therapy (defined as the time from the first dose of IV VPA to the first dose of oral VPA) from the dataset. No statistically significant difference among the three groups was found, indicating that baseline differences in prior IV VPA exposure did not confound the comparison of transition outcomes (Table 3).

### 3.3. Daily Dose of VPA

In this study, the daily doses of VPA (including both intravenous and oral forms) before, during, and after the transition period, were analyzed for all 164 patients included in the study (Table 4).

For the intravenous daily dose, the median daily dose was 1076.50 mg/d for 0 h IV-to-oral transition group, 1200 mg/d for the 0–48 h group, and 1438 mg/d for the >48 h group. There was a statistically significant difference in the intravenous daily doses among the three groups (*p* < 0.001). Post-hoc analysis using the Bonferroni method revealed significant differences between the >48 h group and the 0 h group, as well as between the 0–48 h group and the 0 h group. These results indicated that patients with lower daily doses of VPA were more likely to undergo an immediate switch to oral VPA following the discontinuation of intravenous VPA.

The “transition period” was not applicable for the 0 h IV-to-oral transition group. However, the 0–48 h group reached a daily dose of 2200 mg/d, and the >48 h group reached 2374.50 mg/d during the transition period. Statistical analysis using the Mann–Whitney test revealed significant difference in the daily dose during the transition between the two groups (*p* = 0.01). This finding suggested that the >48 h transition strategy was associated with higher daily dose of VPA during the transition period.

After switching to oral VPA, all three groups maintained a median daily oral dose of 1000 mg/d, with no significant difference among the groups (*p* = 0.25).

### 3.4. Clinical Efficacy

The clinical efficacy was evaluated and analyzed as we described in the Section 2: Materials and Methods. Firstly, the total efficacy rates were calculated as described above: the lowest was found in the 0 h group (90.00%), higher in the 0–48 h group (96.77%) and in the >48 h group (95.16%). Further, a significant difference regarding the clinical efficacy was observed among the three groups (*p* = 0.001) (Table 5). The >48 h group showed the highest rate of clinical efficacy, which was significantly greater than that of the 0 h group (*p* = 0.003) and 0–48 h group (*p* = 0.004). Additionally, the recurrence/exacerbation rates of seizures among the three groups during and after transition were also presented (Table 6).

### 3.5. Blood Plasma Concentrations of VPA

For the clinical use of ASMs, therapeutic drug monitoring (TDM) plays a critical role. In this study, the average blood plasma concentrations of VPA (CVPA) were analyzed separately before, during, and after the IV-to-oral transition. The results indicated no significant differences in CVPA across the three periods (Table 7). Notably, no patients in the 0 h group exceeded a blood plasma concentration of 100 mg/L either before or after the transition. However, during the transition, in the 0–48 h and >48 h groups, five patients (5/62) and twelve patients (12/62) separately exceeded the threshold.

These findings suggest that the >48 h transition strategy requires rigorous monitoring due to the increased likelihood of exceeding the therapeutic range and possible greater fluctuations.

### 3.6. Adverse Drug Reactions

A total of 139 ADRs were reported across the three groups: 30 in the 0 h group, 42 in the 0–48 h group, and 67 in the >48 h group, with the causality rated as “possible” or “probable”, according to the Naranjo Scale. The reported ADRs affected multiple systems, including the hematological, neurological, and digestive systems (Table 8).

Among these, hemoglobin reduction showed statistically significant differences among the groups, with 7 cases in the 0 h group, 9 cases in the 0–48 h group, and 23 cases in the >48 h group (*p* = 0.028). Post-hoc analysis using the Bonferroni method revealed a significantly higher rate of hemoglobin reduction in the >48 h group compared to the other 2 groups.

Headache and dizziness were reported in 5 cases in the 0 h group, and 1 case each in the 0–48 h and >48 h groups (*p* = 0.021). Post-hoc Bonferroni analysis indicated that the rate of headache and dizziness was significantly higher in the 0 h group compared with the other two groups.

Additionally, ALT elevation was reported in 4 cases in the 0 h group, 7 in the 0–48 h group, and 18 in the >48 h group (*p* = 0.012). Bonferroni analysis confirmed that the >48 h group had a significantly higher rate of ALT elevation compared to the other groups.

However, for digestive and skin-related ADRs, no significant differences were found among the groups.

These findings suggest that longer transition times are possibly associated with increased risks of ADRs. Therefore, close monitoring of VPA dosage and transition duration, along with regular blood tests for hematological and liver function indicators, is essential to minimize the risk of adverse reactions during VPA treatment.

## 4. Discussion

VPA injection at a dose of 20–30 mg/kg is recommended as a temporary replacement of oral daily use, administered through either four divided intravenous infusions or as a continuous infusion over 24 h. When rapid achievement and maintenance of effective blood concentration are required, VPA should be administered as a slow intravenous push at a dose of 15 mg/kg, lasting for at least 5 min, followed by an intravenous infusion at a rate of 1 mg/kg/h to rapidly reach a plasma concentration of approximately 75 mg/L. Once the intravenous infusion is discontinued, immediate oral administration is necessary to maintain the desired blood plasma drug concentration. The recommended conventional daily dose for oral VPA ranges from 1000 to 2000 mg, which is ~20–30 mg/kg. If seizures remain difficult to control, the dose can be increased up to 2500 mg/day. For adults with SE, the intravenous dose is commonly 20–40 mg/kg, infusing over more than 10 min, followed by a continuous 1–2 mg/(kg·h) [12]. Once seizures are controlled, the patients should switch to oral therapy (as sequential therapy) for long-term seizure management.

This study retrospectively analyzed epilepsy inpatients who received VPA injection and transitioned to oral treatment before discharge. We investigated the current transition paradigms used for the IV-to-oral transition of VPA, comparing the efficacy and safety of different transition strategies. The total efficacy rates were calculated and presented: the lowest was found in the 0 h group (90.00%), higher in the 0–48 h group (96.77%) and in the >48 h group (95.16%). A significant difference regarding the clinical efficacy was observed among the three groups (*p* = 0.001) (Table 5). The >48 h group showed the highest rate of clinical efficacy, which was significantly greater than that of the 0 h group (*p* = 0.003) and 0–48 h group (*p* = 0.004). However, there was no significant difference for the recurrence/exacerbation rates of seizures among the three groups during and after transition (Table 6).

### 4.1. Analysis of Daily Doses

Our results indicated that the daily doses during the transition period were significantly higher in the 0–48 h group and the >48 h group compared to before and after the transition, reaching 2200 mg/day and 2374.50 mg/day, respectively. Taking an ideal body weight of 60 kg as an example, the median daily doses of VPA in the 0–48 h group and the >48 h group were 36.67 mg/kg and 39.58 mg/kg, respectively, which are higher than the recommended standard daily dose. These results suggest that during the transition period, the daily dose of VPA increases significantly, potentially leading to an increased risk of ADRs. Therefore, the doses of both intravenous and oral VPA during the transition period may need to be adjusted, and further research is needed to provide more evidence for these adjustments.

### 4.2. Analysis of Blood Plasma Concentration

Therapeutic drug monitoring has been attached increasing importance in recent years. Prolonged blood concentrations below the effective therapeutic level may lead to drug tolerance, rendering the previously effective doses ineffective and necessitating dose escalation or a switch to alternative medications. Conversely, if the blood plasma concentration exceeds the therapeutic window, the risk of ADRs increases. These may include liver dysfunction, hematological and lymphatic system abnormalities, gastrointestinal and neurological damage. In severe cases, it may even lead to liver failure, severe bone marrow suppression, fatal pancreatitis, or vasogenic cerebral edema. Therefore, both clinical guidelines and product inserts recommend monitoring VPA blood concentrations to ensure its efficacy and safety. Typically, the recommended therapeutic range for VPA is 50–100 mg/L.

In this study, we retrospectively analyzed the blood concentrations of VPA before, during, and after the transition from intravenous injection to oral tablets. We found that, in both the 0–48 h group and >48 h group, a few cases had blood concentrations exceeding 100 mg/L, while the majority of cases remained below this threshold. Notably, a higher proportion of patients in the >48 h group (19.35%) had blood concentrations exceeding 100 mg/L compared with the 0–48 h group (8.06%), which may be associated with the significantly higher daily doses of VPA administered in the >48 h group. Given that blood concentrations above 100 mg/L may increase the risk of ADRs of VPA, it is essential to be cautious about high doses during the transition. Blood concentrations should be monitored regularly, and the dose of VPA should be adjusted promptly to mitigate the risk of ADRs.

However, in clinical practice, standardized monitoring of VPA blood concentrations remains lacking. In this retrospective study, data on blood concentration monitoring were often incomplete. Specifically, only a few cases strictly followed the trough concentration sampling standard (within 30 min before administration), and only a limited number had complete sequences of intravenous and oral drug concentrations. Additionally, there was a lack of dynamic monitoring at critical timepoints, such as 24 h and 72 h after the transition to oral administration. These data limitations directly hinder the development of a population pharmacokinetic (PopPK) model. Therefore, there is an urgent need for collaboration with clinicians to conduct prospective, multicenter, randomized controlled studies. Meanwhile, this should involve setting up rigorous blood sampling schedules (e.g., trough concentrations at 0.5 h, 2 h, 4 h after the first oral dose, and on the day 3 and 5), and closely monitoring VPA blood concentrations. PopPK modeling should be performed to establish oral dose adjustment formulas, thereby providing more precise medication guidance for clinicians.

### 4.3. Analysis of Adverse Events

Although VPA is generally well-tolerated, it can cause a range of ADRs, some of which can be serious. Accumulating evidence supports that increasing the VPA dose may result in increasing risks and severity of ADRs. For example, as early as the 1990s, Beydoun et al. compared the safety of high doses (blood concentration 555–1040 µmol/L, 80–150 µmol/mL) with low doses (blood concentration 175–345 µmol/L, 25–50 µmol/mL) of VPA [13]. Their findings indicated that ADRs occurred significantly more frequently in the high dose group, including tremors, thrombocytopenia, alopecia, etc.

In this study, we evaluated the safety of the VPA IV-to-oral transition process in 164 epilepsy patients. A total of 70 cases of hematological ADRs were identified [14], including thrombocytopenia and reductions in hemoglobin, with 39 cases showing a decrease in hemoglobin of more than 20%. Apart from hematological issues, liver dysfunction (29 cases) was also commonly observed. Since VPA is primarily metabolized in the liver, its metabolites can be toxic to hepatocytes, leading to liver dysfunction and elevated liver enzymes [15]. Moreover, during the transition process, other common ADRs were also noted, such as allergic reactions, drowsiness, headache, nausea, and vomiting. In conclusion, regular blood tests to monitor complete blood count and liver function, along with close monitoring of neurological, gastrointestinal, and dermatological systems, are essential during VPA therapy to minimize the risk of serious adverse reactions.

When comparing ADR occurrences in the three transition regimens, the >48 h group exhibited a higher incidence of ADRs than the 0 h and 0–48 h groups. Notably, significant differences were observed in hemoglobin reduction and elevated liver enzymes. This may be attributed to the higher daily doses of VPA administered in the >48 h group during different stages of the transition, where the daily dose often exceeded the recommended standard, resulting in higher blood concentrations. These findings suggest that a prolonged transition period correlates with higher the daily dose of VPA and an elevated risk of ADRs.

Therefore, optimizing the IV-to-oral transition regimen for VPA is crucial. A well-balanced approach—avoiding unnecessarily prolonged transitions, ensuring appropriate dose adjustments, and closely monitoring VPA blood plasma concentrations—should be implemented to maximize efficacy while minimizing the risk of adverse effects. Although the >48 h group showed a potential advantage in clinical efficacy over the other transition strategies, its overall suitability remains questionable due to safety concerns. In clinical practice, the choice of transition strategy should be individualized, carefully balancing the potential benefits and risks based on each patient’s specific condition.

### 4.4. Limitations

This study has several limitations that should be considered when interpreting its findings, primarily related to study design, data quality, and outcome assessment.

Firstly, the retrospective study design introduces inherent constraints. Due to its reliance on pre-existing electronic medical records and hospital databases (rather than prospective data collection), we faced challenges with selection bias, incomplete data, and limited control over confounding variables. For selection bias, the study cohort was restricted to inpatients at a single center (Sir Run Run Shaw Hospital) between 2022 and 2023, with enrollment dependent on retrospective identification of patients meeting inclusion criteria—this non-random selection may limit the generalizability of results to other institutions. In addition, in retrospective practice, TDM was not performed according to predefined pharmacokinetic principles—specifically, it lacked systematic rigorous scheduling aligned with VPA’s dosing timelines (e.g., trough concentration sampling before the next intravenous or oral dose) or its elimination half-life (8–15 h in adults). This temporal misalignment means VPA plasma concentration data cannot be reliably linked to outcomes such as seizure recurrence or ADR incidence (e.g., we could not confirm if events occurred when VPA levels were within the therapeutic window [50–100 mg/L] or outside it), precluding meaningful analysis of concentration-dependent efficacy or safety.

Moreover, since ADR assessment was susceptible to confounding and information bias, identifying drug-induced ADRs retrospectively is inherently complex, as multiple factors—including patients’ underlying diseases, disease severity, concurrent use of other medications, and environmental influences—could confound the attribution of events to VPA. Further, information bias (including reporting bias) may have occurred: clinicians and nursing staff were more likely to document favorable outcomes (e.g., successful seizure control) or severe ADRs, while mild ADRs or treatment failures may often be underdocumented in medical records. This imbalance may have overestimated treatment benefit and underestimated safety risks, compromising the objectivity of ADR-related findings. Last but not least, the relatively small sample size (n = 164) may also reduce the statistical power.

## 5. Conclusions

This study evaluated the efficacy and safety of different transition strategies from intravenous to oral VPA in epilepsy management. The findings indicate that a gradual transition may lead to better seizure control and greater stability compared to an abrupt switch. However, extending the transition period beyond 48 h was associated with higher daily valproate doses, possibly greater fluctuations in plasma drug concentrations, and an increased risk of ADRs. To optimize patient safety, careful dose adjustments, regular monitoring of plasma drug levels and when necessary, proactive management of potential ADRs associated with elevated VPA levels are essential.

## Figures and Tables

**Table 1:** Naranjo’s ADR probability scale.

To Assess the ADR, Please Answer the Following Questionnaire and Give the Pertinent Score.				
1. Are there previous conclusive reports on this reaction?	Yes	No	Do not know	Score
2. Did the adverse event appear after the suspected drug was administered?				
3. Did the adverse reaction improve when the drug was discontinued or a specific antagonist was administered?				
4. Did the adverse reaction reappear when the drug was re-administered?				
5. Are there alternative causes (other than the drug) that could on their own have caused the reaction?				
6. Did the reaction reappear when a placebo was given?				
7. Was the drug detected in the blood (or other fluids) in concentrations known to be toxic?				
8. Was the reaction more severe when the dose was increased, or less severe when the dose was decreased?				
9. Did the patient have a similar reaction to the same or similar drugs in any previous exposure?				
10. Was the adverse event confirmed by any objective evidence?				
	Total score			

**Table 2:** Demographic and clinical characteristics of the epilepsy patients included in the study.

	n	Transition Time			χ^2^	*p*
		0 h	0~48 h	>48 h		
**Sex**						
Male	52	16 (40.00)	19 (30.65)	17 (27.42)	1.829	0.401
Female	112	24 (60.00)	43 (69.35)	45 (72.58)		
**Age, years**						
<65	95	25 (62.50)	33 (53.22)	37 (59.68)	0.983	0.612
≥65	69	15 (37.50)	29 (46.78)	25 (40.32)		
**Previous history of epilepsy**						
No	112	27 (67.50)	45 (72.58)	40 (64.52)	0.946	0.623
Yes	52	13 (32.50)	17 (27.42)	22 (35.48)		
**Seizure type**						
GS	87	20 (50.00)	33 (53.23)	34 (54.84)	0.230	0.891
FS	77	20 (50.00)	29 (46.77)	28 (45.16)		

Values are expressed as n (%). GS, generalized seizure; FS, focal seizure.

**Table 3:** Length of IV VPA before switching to oral treatment.

Transition Time	Length of IV VPA Before Switching/h	
0 h	168.26 ± 30.988	*p* > 0.05
0–48 h	121.61 ± 25.835	
>48 h	140.92 ± 36.743	

Values are expressed as mean ± SEM. One-Way ANOVA.

**Table 4:** Daily dose of VPA among 0 h, 0–48 h, and >48 h groups before, during, and after transition.

Transition Time	Daily Dose/mg		
	Intravenous	Transition Period	Oral
0 h	1076.50 (400.00, 1200.00) *#	NA	1000 (1000.00, 1000.00)
0–48 h	1200 (1129.50, 1536.00)	2200 (1875.00, 2462.50)	1000 (800.00, 1000.00)
>48 h	1438 (1199.00, 1536.00)	2374.50 (2111.50, 2694.00) *	1000 (1000.00, 1200.00)
*p*	**<0.001**	**0.01**	0.25

Values are expressed as medians (P25, P75). Bold text indicates statistical significance (*p* < 0.001, Krustal–Wallis tests, followed by Bonfferoni; *p* = 0.01, Mann–Whitney tests). For the intravenous daily dose, *# indicate significant differences between the >48 h group and the 0 h group, as well as between the 0–48 h group and the 0 h group. During the transition period, * indicates a significant difference between 0–48 h and >48 h group.

**Table 5:** Evaluation of clinical efficacy among 0 h, 0–48 h, and >48 h groups.

Transition Time	Daily Dose/mg					
	n	Marked Efficacy	Efficacy	Inefficacy	χ^2^	*p*
0 h	40	15 (37.50)	21 (52.50)	4 (10.00)	18.311	**0.001**
0–48 h	62	25 (40.32)	35 (56.45)	2 (3.23)		
>48 h *#	62	44 (70.97)	15 (24.19)	3 (4.84)		

Values are expressed as n (%). Bold text indicates statistical significance (*p* = 0.001, χ^2^ tests followed by post-hoc analysis using Bonfferoni). *# indicate significant differences between the >48 h group and the 0 h group (*p* = 0.003), as well as between the >48 h group and the 0–48 h group (*p* = 0.004).

**Table 6:** Recurrence/exacerbation rate of seizures 0 h, 0–48 h, and >48 h groups.

Transition Time	Recurrence/Exacerbation Rate		
	n	During Transition	After Transition
0 h	40	NA	5 (12.5)
0–48 h	62	3 (4.84)	3 (4.84)
>48 h	62	2 (3.23)	3 (4.84)

Values are expressed as n (%).

**Table 7:** The mean blood plasma concentrations of VPA among the 0 h, 0–48 h, and >48 h groups.

Mean Blood Plasma Concentrations (mg/L)	Transition Time		
	0 h	0–48 h	>48 h
Pre transition	54.24 ± 24.855	57.78 ± 22.792	63.16 ± 31.972
During transition	NA	77.51 ± 25.384	84.79 ± 28.733
Post transition	48.16 ± 22.855	51.52 ± 23.056	54.39 ± 33.521

Values are expressed as mean ± SEM.

**Table 8:** Adverse drug reactions among 0 h, 0–48 h, and >48 h groups.

Adverse Events		n	Transition Time			χ^2^	*p*
			0 h	0–48 h	>48 h		
Hematologic system	Thrombocytopenia	31	8	11	12	0.094	0.954
	Hemoglobin Reduction	39	7	9	23 #*	7.147	**0.028**
Nervous system	Somnolence	10	0	7	3	5.322	0.051
	Headache/Dizziness	7	5 #*	1	1	6.662	**0.021**
	Mood Disorders	4	1	2	1	0.605	1
Digestive system	Abdominal Pain/Diarrhea	1	1	0	0	3.119	0.21
	Nausea/Vomiting	2	0	1	1	0.721	1
Skin and appendage	Rash/Pruritus	16	4	4	8	1.469	0.48
Others	Elevated liver enzymes	29	4	7	18 #*	8.849	**0.012**

Values are expressed as n. Bold text indicates statistical significance (*p* = 0.028, *p* = 0.021, *p* = 0.012, Fisher’s exact tests followed by followed by post-hoc analysis using Bonfferoni). #* indicate significant differences between the >48 h group and the 0 h group, as well as between the >48 h group and the 0–48 h group both for “Hemoglobin reduction” and “Elevated liver enzymes”. #* also indicates significant differences between the 0 h group and the 0–48 h group, as well as between the 0 h group and the >48 h group for “Headache/Dizziness”.

## Data Availability

The datasets used and/or analyzed during the current study are available from the corresponding author on reasonable request.

## Abbreviations

The following abbreviations are used in this manuscript:

VPA	Valproic acid
CNS	Central neurous system
ASM	Anti-seizure medication
IV	intravenous
SE	Status epilepticus
ADR	Adverse drug reaction
